# Malaria research supported with open access data

**DOI:** 10.1016/j.dib.2022.108009

**Published:** 2022-03-02

**Authors:** Nicholas A. Pullen, Emma Bertran

**Affiliations:** School of Biological Sciences, University of Northern Colorado, Greeley, CO, United States

In a new age of enhanced attention to pandemics, infectious diseases, and science literacy [1] spurred on by the SARS-CoV-2 global threat, some might have missed the news of considerable advances against other diseases and the significant, broad impact of such progress. For example, in November 2021 the positive results of a vaccine against Valley Fever (in canines) were published – the first such successful action targeting a fungus [2]. Even more groundbreaking, a month prior, on October 6, 2021, the World Health Organization (WHO) published recommendations endorsing use of the RTS,S vaccine for malaria in children residing in endemic, high-transmission regions, specifically sub-Saharan Africa [3]. According to the WHO there were nearly a quarter billion malaria cases and over 600,000 malaria-related deaths in 2020, thus representing substantial public health and life quality burdens [4]. While the efficacy of the RTS,S vaccine has been described as modest, there is agreement from modeling studies that this intervention has the potential to prevent millions of cases and tens of thousands of deaths per year [5,6]. Nevertheless, there is much opportunity to reduce the malaria burden through further vaccine development in addition to methods such as mosquito biologic modification, antimalarial drug discovery, and other public health interventions (*e.g.*, netting, avoidance, etc.) [7]. RTS,S should not be viewed as an end-all, as treating malaria has proven to be a fickle endeavor due in part to parasite drug resistance and a complex life cycle. The open sharing of data is a key factor in supporting the swift, reproducible development of additional strategies mitigating the malaria health crisis. This is the motivation for us, the editorial staff, at *Data in Brief* (*DIB*) to highlight data papers concerning malaria recently published in the journal. We hope to encourage further submissions along these lines and look forward to future growth in this and other fields with “special issues” focusing on topics of broad, global importance. Our mission at *DIB* is to support the open discourse and reproducibility inherent to rigorous science.

For the path of inquiry dealing with vaccine development, Lee et al. of the Malaria Vaccine Initiative published a direct submission of mass spectrometry data for Pfs25 [8]. This protein is expressed in the early stages of the lifecycle of new *Plasmodium falciparum* parasites and has been pursued as a vaccine candidate for several decades [9]. However, its detailed structure has proven to be difficult to resolve and bring to bear. With their publication in *DIB*, Lee, et al. provided substantial value in dealing with the disulfide bond distribution in this important target [8].

Another area of robust activity over decades of research has been the discovery, development, testing, and implementation of pharmaceutical inhibitors of plasmodium parasite activity. Of note are the artemisinin-based combination therapies (ACTs), which are considered standard of care in certain settings. Unfortunately, owing in part to their complex life history, malarial parasites have time and again developed resistance to small molecule interventions. This includes developing resistance to ACTs, as observed, for example, over a dozen years ago [10], and more recently highlighted by the WHO in their 2021 annual report [11]. These drugs are still important treatment courses and continued surveillance for resistance is necessary. Sitali et al. published powerful surveillance data on certain indicators of several classes of antimalarials, with their observations indicating that ACTs are still effective in their surveyed population (Zambia) [12,13]. Epidemiological parameters beyond drug resistance checks are an important facet too, and these should include basic local population observations [14] and other qualitative or categorical observations of severe complications such as cerebral malaria [15]. We look forward to continued advances in these areas including novel angles of research in diagnostics development and big data querying code; we also emphasize the importance of searching widely for candidate antimalarials. The ACTs are natural product derivatives, and we are amidst a renaissance of investigation into other plant, fungal, and prokaryotic derived compounds for a variety of conditions, with room for similar studies in the malaria space.

Of course, much effort is appropriately placed in malaria vector research as this prevents the onset of human pathology [16]. Featured examples in this line of investigation include the work of Sikulu et al. who identified age associate markers in *Anopheles* species [17], in support of their larger work that also compared these changes with some degree of overlap in the main dengue fever vector, *Aedes aegypti* [18]. This type of research directly informs advances in vector surveillance in the wild. Organ and tissue specific characterizations of mosquitoes have been an intense area of study, given the potential to better understand – and thus engineer – mosquito life cycle and parasite interactions. Such studies seek to inform mosquito seeking behaviors through characterizing neural proteomes [19] and antennae [20], as well as the resident tissue of the parasite, the midgut [21].

This is not intended to be an exhaustive list, and we apologize for not including other important datasets due to space limitations. We merely wish to highlight *DIB* as a place for open data regarding a very important disease in need of further treatment research. *Data in Brief* welcomes submissions in a variety of contexts (link) including broadly applicable datasets, negative data, and rigorous experiments testing reproducibility of previous research. Regardless of source or type, all data submitted for publication should be of substantive statistical power. We look forward to future submissions in the malaria field that support the development of additional therapies in the arsenal against this deadly disease.

## CRediT authorship contribution statement

**Nicholas A. Pullen:** Writing – original draft, Writing – review & editing. **Emma Bertran:** Writing – review & editing, Data curation.

## Declaration of Competing Interest

None.
